# Morphogenesis and Optoelectronic Properties of Supramolecular Assemblies of Chiral Perylene Diimides in a Binary Solvent System

**DOI:** 10.1038/s41598-017-05692-4

**Published:** 2017-07-14

**Authors:** Xiaobo Shang, Inho Song, Hiroyoshi Ohtsu, Jiaqi Tong, Haoke Zhang, Joon Hak Oh

**Affiliations:** 1 Department of Chemical Engineering, Pohang University of Science and Technology (POSTECH), Pohang, 790-784 South Korea; 2 Department of Chemistry, School of Science, Tokyo Institute of Technology, Tokyo, 152-8550 Japan; 3 Department of Polymer Science and Engineering, Zhejiang University, Hangzhou, 310027 China; 4 Department of Chemistry, Hong Kong University of Science & Technology, Hong Kong, China

## Abstract

Chiral supramolecular structures are attracting great attention due to their specific properties and high potential in chiral sensing and separation. Herein, supramolecular assembling behaviors of chiral perylene diimides have been systematically investigated in a mixed solution of tetrahydrofuran and water. They exhibit remarkably different morphologies and chiral aggregation behaviors depending on the mixing ratio of the solvents, i.e., the fraction of water. The morphogenesis and optoelectronic properties of chiral supramolecular structures have been thoroughly studied using a range of experimental and theoretical methods to investigate the morphological effects of chiral supramolecular assemblies on the electrical performances and photogenerated charge-carrier behaviors. In addition, chiral perylene diimides have been discriminated by combining vibrational circular dichroism with theoretical calculations, for the first time. The chiral supramolecular nanostructures developed herein strongly absorb visible spectral region and exhibit high photoresponsivity and detectivity, opening up new opportunities for practical applications in optoelectronics.

## Introduction

Chirality is a basic characteristic of living matter and the natural environment. Currently, separation and detection of chiral materials are mostly carried out using off-line analytical techniques, such as chromatography. Other methods, including femtosecond pulses^1^, microwaves^2, 3^, photoionization^4^, superchiral light^5^, and polarimetry^6^ have also been used to determine chirality. Compared with the former methods, vibrational circular dichroism (VCD) is much simpler yet more powerful for absolute chiral discrimination^7^. VCD is defined as the difference in the absorbance of a chiral molecule for left circularly polarized (LCP) versus right circularly polarized (RCP) irradiation during vibrational excitation and extends into the infrared and near-infrared ranges. VCD can provide three-dimensional structural information because of its sensitivity to the mutual orientation of distinct molecular groups. Density functional theory (DFT) was first applied to the calculation of VCD spectra in the early 1990s, and VCD has subsequently been widely used to determine absolute configurations (ACs)^8–10^.

Chirality is universal and can be observed at various hierarchical levels, from the subatomic and molecular to supramolecular, nanoscopic, macroscopic, and galactic scales^11^. Among these various levels, chiral molecules at the supramolecular level are of great importance because they are highly relevant to physics, biology, chemistry, materials science, and nanoscience^12^. In particular, π-conjugated chiral molecules, such as perylene diimides (PDIs), have attracted special interest because they can easily assemble into chiral supramolecular nanostructures^13–16^. Besides, the molecular ordering and kinetics of rylene diimides have been reported previously^17–22^. Nonetheless, research on chiral nanostructures is still in its infancy^23^, especially for organic chiral nanomaterials.

We recently observed the amplified chirality of chiral perylene diimides bearing 1-phenylethylamine (CPDI-Ph) nanowires fabricated by a non-solvent mediated nucleation method, compared with the molecular chirality, and utilized this unique feature for high-performance chiroptical sensing^24^. Herein, we have systematically investigated the morphogenesis of chiral supramolecular self-assemblies of CPDI-Ph in different aggregation states using a binary solvent system of tetrahydrofuran (THF) and water. Their dynamic aggregation processes have been analyzed by time-dependent *in-situ* UV-visible (UV-vis) spectroscopy, circular dichroism (CD) spectroscopy, and scanning electron microscopy (SEM). Due to the polarity of the solvent and the hydrophobic effect, the binary solvent system has yielded different morphologies depending on the fraction of water (*f*_w_), which correspond well to their differing performance in organic field-effect transistors (OFETs). In addition, we have also utilized VCD to effectively discriminate the chiral PDI derivatives.

## Results and Discussion

### Aggregation and chirality study of CPDI-Ph in THF/water system

**Figure 1:**
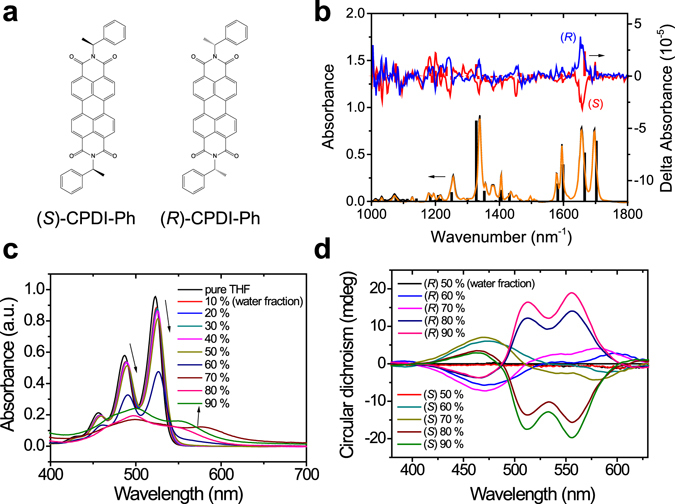
(**a**) Molecular structures of (*S*)-CPDI-Ph and (*R*)-CPDI-Ph. (**b**) Experiment and Calculation results of IR and VCD spectra of CPDI-Ph. Solid lines show experiment data of CPDI-Ph and blocks show calculation results for (*R*)-CPDI-Ph. (**c**) UV-vis spectra of (*S*)-CPDI-Ph and (**d**) CD spectra of enantiomeric CPDI-Ph in THF/water mixed solution (1.0 × 10^−5^ M) with different *f*_w_.

The chiral *n*-type semiconductors (*R*)-CPDI-Ph and (*S*)-CPDI-Ph (Fig. 1a) were synthesized from perylene-3,4,9,10-tetracarboxylic dianhydride and (*R*)- and (*S*)-1-phenylethylamine, respectively. The UV-vis and photoluminescence (PL) spectra of CPDI-Ph solutions in THF (1.0 × 10^−5^ M) are illustrated in Figure S1a. For the two enantiomers, the absorption spectra in solution were similar, with four peaks at 527, 490, 460, and 435 nm, corresponding to the 0–0, 0–1, 0–2, and 0–3 transitions of well-resolved vibronic structures of individual CPDI-Ph molecules, respectively. CD spectroscopy, which measures the differential absorption of LCP and RCP light, provides structural information for the ground electronic state of a system^25^. In dilute solutions of CPDI-Ph, there was no CD signal for wavelengths longer than 400 nm, indicating there was no chirality transfer from the chiral pendent to the PDI core (Figure S1b). To better understand the nature of the observed chirality, VCD measurements were carried out in a chloroform–d solution of CPDI-Ph (21.7 mmol L^−1^). The two isomers exhibited mirror images in the VCD spectra (Fig. 1b), and the peak wavenumber matched the corresponding infrared spectra. Positive and negative peaks corresponding to ring stretching at 1,337 and 1,653 cm^−1^ were observed for (*R*)-CPDI-Ph and (*S*)-CPDI-Ph isomers, respectively. The peaks between 1,640 and 1,740 cm^−1^ were attributed to imide C=O stretching. We also calculated the VCD spectra via the DFT method (B3LYP/6–311 + G**) using Gaussian 09. The main peaks from VCD calculations were in good agreement with the experimental results, leading to the facile identification of the AC. To the best of our knowledge, this is the first demonstration of the discrimination of semiconducting chiral PDIs by VCD.

In general, different aggregation states have different spectral features; thus, the aggregation behaviors of the CPDI-Ph were investigated by monitoring changes in the UV-Vis, PL, and CD spectra. THF was chosen as a good solvent, and water as a non-solvent. In the THF–water system, the UV-vis and CD spectra were nearly unchanged when the *f*_w_ increased from 0 to 50%, indicating the monomeric state (Figs 1c,d and S2a). The aggregation behaviors were confirmed by monitoring the color change of solutions. At a large amount of water (i.e., *f*_w_ > 50%), the color of solution was changed from yellow to red due to the aggregation phenomena. When the *f*_w_ reached 60%, the absorption features changed dramatically in the UV-vis spectra, and a strong bisignated Cotton effect was observed in the CD spectra with positive and negative maxima at 470 and 600 nm for (*S*)-CPDI-Ph, respectively. Aggregation generally leads to the Cotton effect, indicating an aggregation-induced CD (AICD) effect^26–28^. CD provides information on the overall molecular stereochemistry (conformation and configuration)^29^. Peaks longer than 400 nm are thought to stem from the PDI core, implying the successful chirality transfer from the 1-phenylethylamine pendants to the PDI moieties in the aggregated state. PL spectra of CPDI-Ph in the THF–water system demonstrated an aggregation-induced quenching effect due to the attractive dipole–dipole interaction and effective intermolecular π–π stacking of dye molecules as *f*_w_ reached 60% (Figure S2b,c). The presence of aggregated structures in the THF–water mixture was also demonstrated by dynamic light scattering (DLS) experiments (Figure S2d). As *f*_w_ increased above 60%, nanoscale aggregates appeared with size dimensions of hundreds of nanometers.

To quantify the propensity of the π-stack formation of CPDI-Ph, a degree of aggregtion (*α*_agg_) was calculated from the solvent-dependent UV-vis and PL spectra (Figure S3). Higher *α*_agg_ values indicate stronger aggregation. An *f*_w_ of 50% was the crucial solvent composition, corresponding to *α*_agg_ of 0.18. The *α*_agg_ sharply increased to 0.4–0.6 at an *f*_w_ of 60% and reached nearly 1 for an *f*_w_ of 70%; these results are due to the different polarities and hydrophobic effects in the mixed THF–water solution (details in methods).

### Time-dependent self-assembly of CPDI-Ph in THF/water system

**Figure 2:**
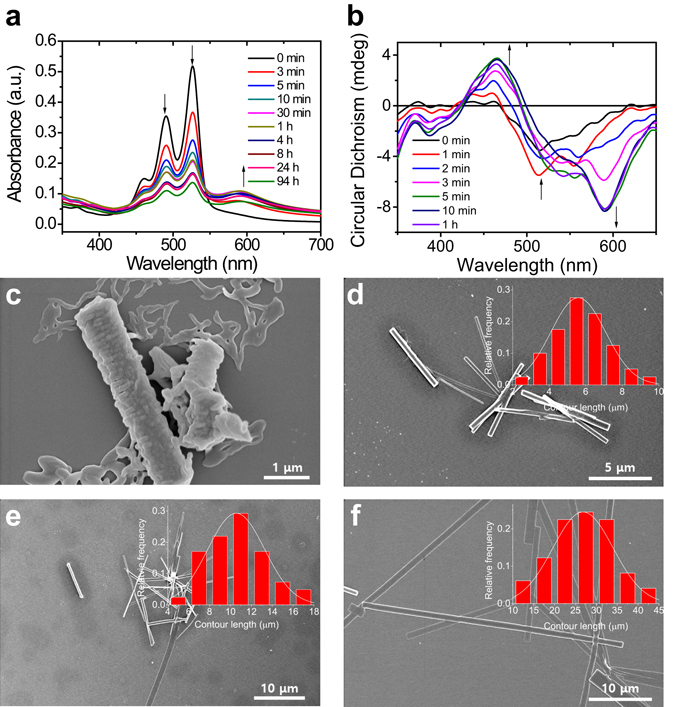
(**a**) Time-dependent UV-vis and (**b**) CD spectra of (*S*)-CPDI-Ph in THF/water mixed solution (1.0 × 10^−5^ M) with *f*_w_ = 60%. SEM images of (*S*)-CPDI-Ph aggregates in THF/water mixed solution (1.0 × 10^−5^ M) with *f*_w_ = 60% after (**c**) 10 s, (**d**) 3 min, (**e**) 10 min, and (**f**) 2 h.

Time-dependent UV-vis, CD, and SEM analyses in the mixed system (i.e., *f*_w_ ≥ 60%) were performed to further investigate the strong aggregation behavior of hydrophobic CPDI-Ph molecules. At an *f*_w_ of 60%, the intensity of the absorption at 490 and 527 nm decreased dramatically with time, and the shoulder peak at 592 nm increased (Fig. 2a). Similar changes were seen for other PDI derivative aggregates, which tended to self-assemble into ordered structures because of the strong π–π interaction between the perylene cores^30, 31^. According to time-dependent SEM analysis at an *f*_w_ of 60%, CPDI-Ph showed metastable nanobelts in freshly sample, forming gradually well-defined nanobelts in 3 min, and the nanobelts elongated over time (Figs 2c–f and S4). After 2 h of self-assembly, substantially elongated aggregates formed. The resulting nanobelts exhibited a five-fold increase in length in comparison with those formed in 3 min, with relatively uniform diameters, indicating epitaxial growth. Longer self-assembly time resulted in one-dimensional nanoassemblies, which are typically observed for PDI derivatives.

When *f*_w_ increased to 90%, the time-dependent UV-vis spectra showed main peaks at 500 and 555 nm (Figure S5a). Time-dependent SEM images confirmed the formation of very small, disordered nanoparticles (Figure S5c). Both the UV-vis spectra and the morphologies were unchanged as time elapsed, indicating the formation of nanoparticles. Interestingly, *f*_w_ values of 70% and 80% yielded dynamic transformations. When the *f*_w_ reached 70%, nanoparticles initially formed with UV-vis peaks similar to those seen at an *f*_w_ of 90% (Figure S6a). However, the intensity of the original peaks at 500 and 555 nm decreased, and a new peak appeared at 575 nm within 3 min. UV-vis spectra remained constant after the transition. The process was further evidenced by time-dependent SEM measurements (Figure S6c–f). We confirmed that nanobelts with an average length of ~1.5 μm, as well as nanoparticles, formed after 5 min. After 4 h of self-assembly, the nanoparticles disappeared completely, while the nanobelts lengthened slightly, by 0.4 μm. The morphology did not change, even after 1 day, implying thermodynamic stability. Compared with the nanobelts formed at an *f*_w_ of 60%, those formed at 70% were shorter and maintained a constant length, indicating different growth routes for the two solutions.

**Figure 3:**
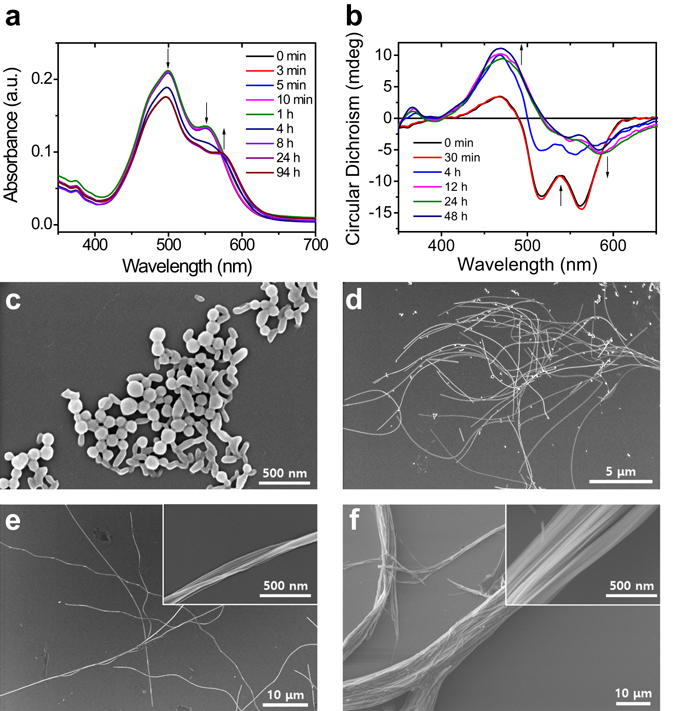
(**a**) Time-dependent UV-vis and (**b**) CD spectra of (*S*)-CPDI-Ph in THF/water mixed solution (1.0 × 10^−5^ M) with *f*_w_ = 80%. Time-dependent SEM images of (*S*)-CPDI-Ph in THF/water mixed solution (1.0 × 10^−5^ M) with *f*_w_ = 80% after (**c**) 30 min, (**d**) 4 h, (**e**) 8 h, and (**f**) 24 h.

The CPDI-Ph spectra for an *f*_w_ of 80% exhibited two peaks at 500 and 555 nm, corresponding to the formation of nanoparticles up to 1 h after water injection (Fig. 3a). After 8 h of self-assembly, the peak at 555 nm disappeared, and a new peak appeared at 575 nm as the nanoparticles disappeared, similar to the transition that occurred for an *f*_w_ of 70%. Time-dependent SEM images (Fig. 3c–f) confirmed this assumption. The initial nanoparticles gradually transformed into extremely long nanofibers within 8 h. After 24 h, the more thermally stable nanofiber bundles were observed. Time-dependent CD also confirmed this hypothesis (Figs 2b and 3b and S5b and S6b). The two main peaks at 516 and 559 nm were unchanged at an *f*_w_ of 90%, indicating the formation of nanoparticles. These two peaks were also observed at *f*_w_ values of 70% and 80% within 3 min and 1 h, respectively. As time elapsed, the two main peaks gradually decreased and transformed into new peaks near 587 nm, indicating the formation of nanobelts and nanofibers at *f*_w_ values of 70% and 80%, respectively.

**Figure 4:**
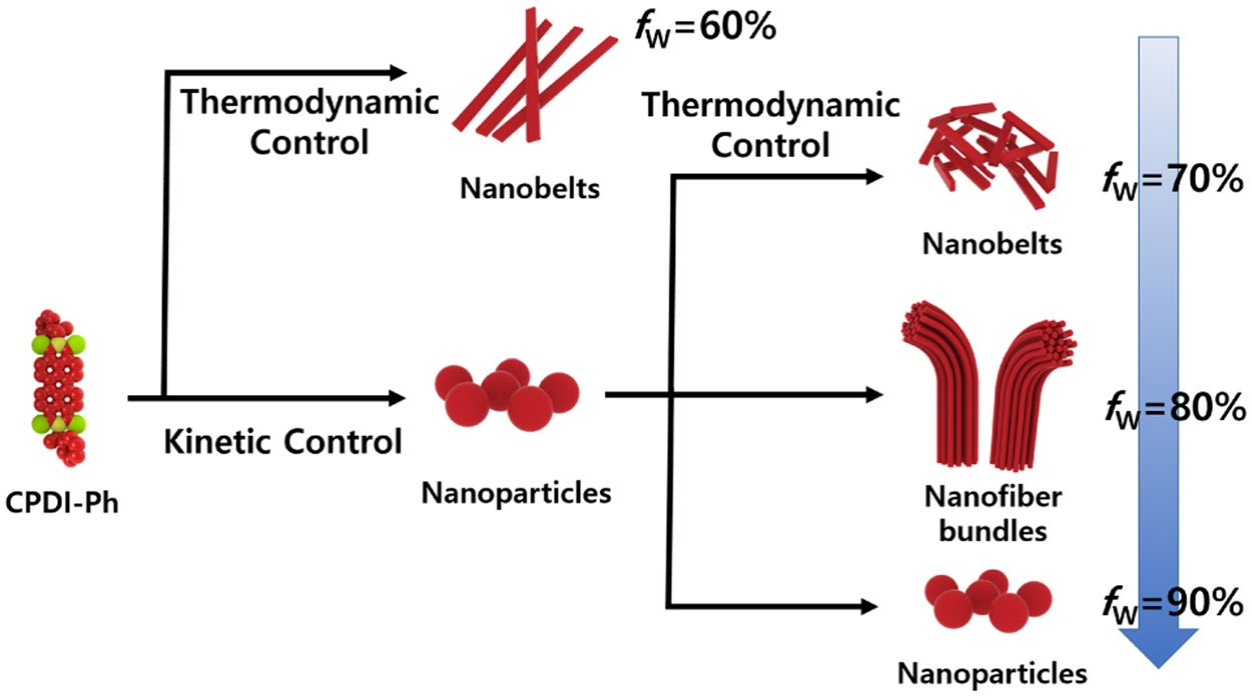
Schematic representation of the morphological transformation of CPDI-Ph.

Based on these findings, we conclude that the polarity of the solvent and the hydrophobic effect may play important roles in the self-assembly of PDIs^32^. Competition between reassembly (thermodynamic control) and fast nucleation (kinetic control) (Fig. 4) may also play a role. At a low *f*_w_, the solvent mixture increased the solvation power than the mixture with a high *f*_w_, so the CPDI-Ph molecules had more accessible mobility to undergo reassembly. As a result, at an *f*_w_ of 60%, thermodynamic factors dominated the self-assembly and preferentially formed highly ordered nanostructures to minimize the free energy of the system. With an increase of water content, solvent became more unfavorable for reassembly, therefore, the CPDI-Ph molecules aggregated too fast to adjust their optimal conformation^31^. Therefore, at an *f*_w_ of 90%, the nucleation dominated the assembly growth, and kinetically favored nanostructures were obtained. For *f*_w_ values of 70% and 80%, initial nanoparticle formation was kinetically favored. Compared with the higher polarity and stronger hydrophobic effect that occurred at an *f*_w_ of 90%, the nanoparticles transformed into more stable nanobelts and nanofiber bundles. At *f*_w_ of 90%, dynamic molecular rearrangements into 1-D assemblies could not take place effectively in the mixed solvent system with such a high polarity and a low hydrophobicity, mostly likely due to the greatly reduced mobility of the chiral molecules. On the other hand, at *f*_w_ of 70% and 80%, the intermediate nanoparticle morphologies were gradually transformed into thermodynamically stable nanobelts and nanofibers, respectively, as shown in Figs 3d and S6d. Therefore, it is considered that the morphological transitions occur via the structural rearrangement mechanism in which the relative polarity and hydrophobicity of the mixed solvent system play a critical role in determining the morphologies^33^.

### Application of nanomaterials of CPDI-Ph in organic phototransistors

**Figure 5:**
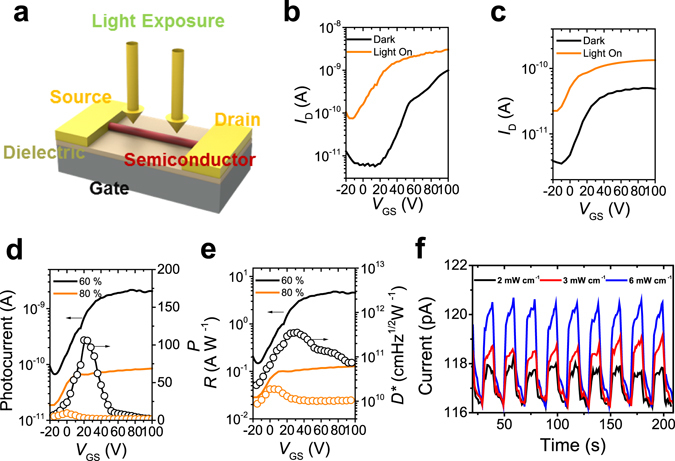
(**a**) Schematic diagram of organic field-effect transistor based on nanomaterials. Transfer curves of (*S*)-CPDI-Ph OPTs based on nanomaterials in THF/water mixed solutions (1.0 × 10^−5^ M) with *f*_w_ of (**b**) 60% (average *W*/*L* = 0.017) and (**c**) 80% (average *W*/*L* = 0.006). (**d**) Photocurrent and photocurrent/dark current ratio of the NM-OPTs. (**e**) Responsivity and detectivity of the NM-OPTs. (**f**) Photo-switching results of nanomaterials in THF/water mixed solution (1.0 × 10^−5^ M) with *f*_w_ = 60% under various intensity of polychromatic light illumination.

The effect of morphology on the electrical conductivity of the self-assembled PDI aggregates was reported previously^34^. The electronic properties of the supramolecular assemblies of *L*-lysine-functionalized 4ClPDI were also related to their molecular packing, which was tailored using various assembly strategies^31^. However, to the best of our knowledge, this is the first reported investigation of the field-effect mobility of OFETs based on the morphology of self-assembled chiral PDI semiconductors. We used aged CPDI-Ph supramolecular nanostructures, prepared at *f*_w_ values of 60% and 80% as active materials in OFETs, to investigate the correlation between nanostructure morphology and optoelectronic properties. We prepared the individual chiral nanomaterial-based organic phototransistors (NM-OPTs) on *n*-octadecyltrimethoxysilane (OTS)-treated SiO_2_/Si substrates (Fig. 5a). Polychromatic light (λ = 450–650 nm) was used as the light source to investigate the photoresponses of the OPTs (Table 1 and Fig. 5b,c). All the enantiomers showed nearly identical electrical properties under the same conditions for CPDI-Ph nanomaterials prepared at *f*_w_ of 60% and 80%. The electron mobilities of the 60%- and 80%-*f*_w_ (*R*)-CPDI-Ph were 3.8 × 10^−3^ and 2.3 × 10^−3^ cm^2^ V^−1^s^−1^ under dark conditions, respectively. When the light illuminated the material, the NM-OPT current increased significantly, while the threshold voltage (*V*_T_) shifted negatively, enabling the device to be turned on more easily. Many photogenerated charge carriers were trapped under the source, thus lowering the source–channel potential barrier. Meanwhile, the photogenerated current increased the total current. The field-effect mobility of 60%-*f*_w_ CPDI-Ph was higher than that of 80%-*f*_w_ CPDI-Ph under both dark and polychromatic light illumination, due to the different nanomaterial morphologies.

**Table 1:** Summary of the performances of (*S*)-CPDI-Ph and (*R*)-CPDI-Ph nanomaterials in THF/water mixed solution (1.0 × 10^−5^ M) with 60% and 80% of H_2_O.

Water Amount	Chirality	Light Illumination	*I*_on_/*I*_off_	*V*_t_ (V)	Mobility (×10^−3^ cm^2^ V^−1^ s^−1^)
60%	(*S*)	Dark	>10^2^	14.7^a^ (±3.8)^b^	3.6^a^ (±0.51)^b^
		Light on	>10	−28.1 (±4.9)	6.4 (±0.89)
	(*R*)	Dark	>10^2^	12.9 (±2.8)	3.8 (±0.69)
		Light on	>10	−27.8 (±5.2)	6.8 (±0.94)
80%	(*S*)	Dark	>10	−15.1 (±3.1)	2.1 (±0.61)
		Light on	<10	−37.9 (±3.5)	4.8 (±0.87)
	(*R*)	Dark	>10	−13.7 (±4.1)	2.3 (±0.68)
		Light on	<10	−39.2 (±3.4)	4.9 (±0.61)

^a^The average values obtained for at least six devices from more than two different batches.

^b^The standard deviation values obtained for at least six devices from more than two different batches.

To quantify the photoresponse of the NM-OPTs, photocurrent, photoresponsivity (*R*), photocurrent/dark current ratio (*P*), and detectivity (D*) were evaluated using transfer characteristics coupled with light irradiation (Fig. 5d,e)^35, 36^. Under polychromatic light illumination, NM-OPTs based on 60%-*f*_w_ CPDI-Ph yielded maximum *P*, *R*, and D* values of 106, 4.62 AW^−1^, and 3.58 × 10^11^ cm Hz^1/2^ W^−1^, respectively. In contrast, NM-OPTs based on 80%-*f*_w_ CPDI-Ph displayed much poorer performance, with maximum *P*, *R*, and D* values of 10, 0.13 AW^−1^, and 1.86 × 10^10^ cm Hz^1/2^ W^−1^, respectively. The stronger hydrophobic effect during the synthesis of 80%-*f*_w_ CPDI-Ph nanomaterials disturbed the molecular ordering, leading to reduced exciton dissociation as a result of charge recombination in the disordered area. On the other hand, 60%-*f*_w_ CPDI-Ph nanomaterials enabled efficient exciton dissociation and charge transport due to their well-ordered molecular packing and yielded higher *P*, *R*, and D* values, as confirmed by powder X-ray diffractions (PXRDs) (Figure S7). The PXRD peak at 2*θ* around 5.8° indicates that the periodic distance is 14.7 Å for 60%-*f*_w_ and 15.3 Å for 80%-*f*_w_. This corresponds to 4 π-π stacking distances: if 4 planes of CPDI core are considered as 1 unit, this unit shows the periodicity. Therefore, in average, the π-π stacking distances are estimated to be ~3.67 Å for 60%-*f*_w_ and 3.82 Å for 80%-*f*_w_, indicating the assembly prepared from 60%-*f*_w_ has denser π-π stacking structure than that obtained from 80%-*f*_w_. In addition, the contact quality between the semiconducting material and source-drain electrodes is an important factor for the performance of optoelectronic devices^31^. The nanofibers from 80%-*f*_w_ is a bit twisted in comparison with nanobelts from 60%-*f*_w_, which may also affect their poor optoelectronic properties.

We also conducted photo-switching tests under various polychromatic illumination intensities using NM-OPTs based on 60%-*f*_w_ CPDI-Ph (Fig. 5f). When several pulses of illumination were applied, the NM-OPTs exhibited light-intensity-dependent photoresponses. These π-conjugated semiconductor systems strongly absorbed visible light and detected the intensity of the illuminated light, which may open the door for practical optoelectronic applications in the visible spectral region.

## Conclusion

In conclusion, we synthesized two chiral semiconductor CPDI-Ph compounds and investigated their chiral supramolecular aggregation behaviors in a THF–water solution system. We found that the morphogenesis of the chiral supramolecular assembly was closely related to the relative competition between thermodynamic and kinetic factors at a given *f*_w_, which was significantly affected by the polarity and hydrophobicity of the mixed solvent. Interestingly, the *in-situ* analysis using time-dependent UV-vis spectra, CD spectra, and SEM images revealed that nanobelts were directly formed at an *f*_w_ of 60%, whereas the morphological transformations from nanoparticles to nanobelts and extremely long nanofiber bundles took place at *f*_w_ of 70% and 80%, respectively. The photoresponsive behaviors of supramolecular nanostructures fabricated at *f*_w_ of 60% and 80% were compared to investigate their structure-property relationships. Well-ordered nanobelts in the 60%-*f*_w_ system exhibited excellent light detection and photo-switching abilities under various light illumination intensities because of their well-ordered molecular packing. To the best of our knowledge, these results are the first demonstration to reveal the effects of the morphologies of chiral PDI supramolecular assemblies on the optoelectronic performances in OPTs. We also discriminated chiral semiconductor PDIs for the first time using both VCD and calculations, demonstrating a facile method for the discrimination of chiral semiconductors. The chiral supramolecular nanostructures developed herein show high photoresponsivity and detectivity in the visible spectral region, which paves a new way for practical applications in optoelectronics.

## Methods

### Materials analysis

The absorption spectra was measured on a Cary 5000 UV-Vis-NIR spectrophotometer for the CPDI-Ph solutions. An SEM image was measured using a Hitachi cold SEM microscope. The CD results were processed using J-815 Spectropolarimeter (JASCO) in a 1 mm quartz cuvette using a step resolution of 0.1 nm, a scan speed of 100 nm min^−1^, a sensitivity of 0.1 nm, and a response time of 0.5 s. PL spectra were recorded on FP-6500 spectrofluorometer (JASCO). The DFT calculations were performed using the Gaussian 09 package with the nonlocal hybrid Becke three-parameter Lee-Yang-Parr (B3LYP) function and the 6–31 G basis set. Vibrational circular dichoism (VCD) was measured using Chiralir (BioTools).

### Device fabrication and optoelectrical measurements

The bottom-gate top-contact NM-OPTs were fabricated with a heavily *n*-doped Si wafers with a 300 nm SiO_2_ dielectric (*C*_i_ = 10 nF cm^−2^). The substrates were treated with *n*-octadecyltrimethoxysilane (OTS) in the solution phase. NWs were drop-cast onto OTS-treated SiO_2_/Si substrate. Gold electrodes (40 nm) were thermally evaporated using a shadow mask. The current-voltage characteristics of the devices and the photoresponses upon on-and-off switching of light were measured in a nitrogen-filled glove box using Keithley 4200-SCS semiconductor parametric analyzer.

### Investigation on the aggregation of CPDI-Ph with UV-vis and PL spectra

To quantify the propensity of π-stack formation for this dye, we estimated the mole fraction of aggregated dyes (*α*_agg_) in the THF-water mixtures from the solvent-dependent UV-visible studies by using the [Eq. (1)]^37^. In this equation, *A*_mix_ is the absorbance at 528 nm for CPDI-Ph at a given solvent, while *A*_mon_ and *A*_agg_ denote the absorbance at 528 nm at the lowest and highest THF/water ratios, respectively. The *α*_agg_ values at various solvent mixtures for the PDI derivatives investigated here are plotted as a function of the solvent composition in Figure S3a. At the fraction of water (*f*_W_) from 50% to 70%, strong aggregation began to occur. For example, the *α*_agg_ reaches almost 1 at 70% (*f*_W_).

1 $${{\boldsymbol{\alpha }}}_{{\boldsymbol{agg}}}\approx ({{\boldsymbol{A}}}_{{\bf{mix}}}-{{\boldsymbol{A}}}_{{\bf{mon}}})/({{\boldsymbol{A}}}_{{\bf{agg}}}-{{\boldsymbol{A}}}_{{\bf{mon}}})$$

Traditional PDIs have near-unity fluorescence yield in dilute solutions, while weakly emissive in aggregated states. This phenomenon is referred to as aggregation-caused quenching (ACQ) effect due to the attractive dipole-dipole interactions and/or effective intermolecular π-π stacking in molecular aggregates^38^. In dilute chloroform solution (1.0 × 10^−5^ M), the PL spectrum of the CPDI-Ph shows stokes shift and has emission peaks at 544 nm and 573 nm with a shoulder at around 624 nm (Figure S1a). This is typical feature of *N*, *N*′-substituted PDIs. To get further understanding of the emission and aggregation properties of the CPDI-Ph, we measured the fluorescence spectra of CPDI-Ph in THF-water with different *f*_w_ values (Figures S2b,c and S3b). From the spectra, we calculated the degree of aggregation (*α*_agg_), which is the fraction of molecules present in the aggregates by using the [Eq. (2)]^39^:

2 $${{\boldsymbol{\alpha }}}_{{\bf{agg}}}\approx ({{\boldsymbol{I}}}_{{\bf{i}}}-{{\boldsymbol{I}}}_{{\bf{0}}})/({{\boldsymbol{I}}}_{{\bf{100}}}-{{\boldsymbol{I}}}_{{\bf{0}}})$$

where *I*_i_ is the emission intensity at a given composition, *I*_0_ the emission intensity detected in pure THF, and *I*_100_ is the emission intensity measured in pure water. Then we plotted *α*_agg_ as a function of the amount of water present in the solvent mixture. The quenching factor *Φ*_q_ is calculated by using the [Eq. (3)]^38^:

3 $${{\boldsymbol{\Phi }}}_{{\bf{q}}}\approx ({{\boldsymbol{\Phi }}}_{{\bf{solut}}}-{{\boldsymbol{\Phi }}}_{{\bf{aggre}}})/{{\boldsymbol{\Phi }}}_{{\bf{solut}}}$$

where *Φ*_solut_ and *Φ*_aggre_ are the emission quantum efficiency of the dye in dilute solution and in the aggregated state, respectively. The quenching factor *Φ*_q_ corresponds to the quantum yield for both (*S*)-CPDI-Ph and (*R*)-CPDI-Ph (Figure S3c and d). In the THF-water mixtures, the emission spectra display monotonous red-shift with addition of water. Meanwhile, the quantum efficiency exhibits continuous dropping with the percentage of water increases from 0% to 50%, indicating that CPDI-Ph is typical ACQ luminogen in the THF-water solvent system. The red-shift of the maximum observation peak from 536 nm to 541 nm and the small decrease of the intensity at *f*_w_ from 0 to 50% can be ascribed to the hydrophilic effect of the mixed solvent.

### Evaluation methods of optoelectrical properties

The field-effect mobility (*μ*) and the threshold voltage (*V*_T_) were estimated in the saturation regime (*V*_DS_ = −100 V) with the following equation:

4 $${{\boldsymbol{I}}}_{{\boldsymbol{D}}}=\frac{{\boldsymbol{W}}}{{\bf{2}}{\boldsymbol{L}}}{\boldsymbol{\mu }}{{\boldsymbol{C}}}_{{\boldsymbol{i}}}{({{\boldsymbol{V}}}_{{\boldsymbol{G}}}-{{\boldsymbol{V}}}_{{\boldsymbol{T}}})}^{{\bf{2}}}$$

where *I*_D_ is the drain current, *W* and *L* are the semiconductor channel width and length, respectively. *μ* is the mobility and *C*_i_ is the capacitance per unit area of the gate dielectric. *V*_G_ and *V*_T_ are the gate voltage and threshold voltage, respectively.

In order to investigate photosensitivity for OPTs, photoresponsivity (*R*) and photocurrent/dark-current ratio (*P*) were calculated from transfer characteristics coupled with light irradiation. The *R* and *P* values are typically defined by the following equations^35^:

5 $${\boldsymbol{R}}=\frac{{{\boldsymbol{I}}}_{{\boldsymbol{ph}}}}{{{\boldsymbol{P}}}_{{\boldsymbol{inc}}}}=\frac{{{\boldsymbol{I}}}_{{\boldsymbol{light}}}-{{\boldsymbol{I}}}_{{\boldsymbol{dark}}}}{{{\boldsymbol{P}}}_{{\boldsymbol{inc}}}}$$

6 $${\boldsymbol{P}}=\frac{{{\boldsymbol{I}}}_{{\boldsymbol{light}}}-{{\boldsymbol{I}}}_{{\boldsymbol{dark}}}}{{{\boldsymbol{I}}}_{{\boldsymbol{dark}}}}$$

where *I*_ph_ is the photocurrent, *P*_inc_ the incident illumination power on the channel of the device, *I*_light_ the drain current under illumination, and *I*_dark_ the drain current in the dark, respectively. Besides *R* and *P*, the specific detectivity (D*) is also one of the key figureof-merits for a photodetector, which usually describes the smallest detectable signal^36, 40^:

7 $${{\boldsymbol{D}}}^{\ast }=\frac{{({\boldsymbol{AB}})}^{{\bf{1}}{\boldsymbol{/}}{\bf{2}}}}{{\boldsymbol{NEP}}}({\boldsymbol{cm}}\,{\boldsymbol{H}}{{\boldsymbol{z}}}^{{\bf{1}}{\boldsymbol{/}}{\bf{2}}}{{\boldsymbol{W}}}^{-1})$$

8 $${\boldsymbol{NEP}}=\frac{{{\boldsymbol{i}}}_{{\boldsymbol{n}}}^{{2}^{{\bf{1}}{\boldsymbol{/}}{\bf{2}}}}}{{\boldsymbol{R}}}({\bf{W}})$$

where *A* is the effective area of the detector in cm^2^, B is bandwidth, NEP is the noise equivalent power, ${i}_{n}^{{2}^{1/2}}$ is the measured noise current and *R* is the responsivity. If the shot noise from the dark current is the major contribution to the noise limiting the detectivity, then the detectivity can be simplified as:

9 $${{\boldsymbol{D}}}^{\ast }=\frac{{\boldsymbol{R}}}{{({\bf{2}}{\boldsymbol{e}}\cdot {{\boldsymbol{I}}}_{{\boldsymbol{dark}}}/{\boldsymbol{A}})}^{{\bf{1}}{\boldsymbol{/}}{\bf{2}}}}$$

In this case, *A* is the total cross-sectional area of the CPDI-Ph NWs; thus, *I*_dark_/*A* gives dark current density. [Eq. (9)] suggests that the high responsivity and the low dark current will naturally lead to high detectivities.

## Electronic supplementary material

Supplementary Information
