# Supplementary material for: Morphogenesis and Optoelectronic Properties of Supramolecular Assemblies of Chiral Perylene Diimides in a Binary Solvent System
Source: Sci Rep. 2017 Jul 14;7:5508. doi: 10.1038/s41598-017-05692-4 (PMC5511204; doi:10.1038/s41598-017-05692-4)
Supplement: Supplementary file 1 — Supplementary Information [file 41598_2017_5692_MOESM1_ESM.pdf]

Supplementary Information:

Morphogenesis and Optoelectronic Properties of Supramolecular Assemblies of Chiral Perylene Diimides in a Binary Solvent System

Xiaobo Shang[1,†], Inho Song[1,†], Hiroyoshi Ohtsu[2], Jiaqi Tong[3], Haoke Zhang[4], and Joon Hak Oh[1,*]

[1]Department of Chemical Engineering, Pohang University of Science and Technology (POSTECH), Pohang, 790-784, South Korea

[2]Department of Chemistry, School of Science Tokyo Institute of Technology, Tokyo, 152-8550, Japan

[3]Department of Polymer Science and Engineering, Zhejiang University, Hangzhou, 310027, China

[4]Department of Chemistry, Hong Kong University of Science & Technology, Hong Kong, China

[*] E-mail: joonhoh@postech.ac.kr

[†] These authors contributed equally to this work

Table of Contents

Supporting Figures

Optical properties of CPDI-Ph in THF solution (Figure S1) ------------------------------------- S3

Optical properties of CPDI-Ph in THF/water mixed solution with different f_w (Figure S2) -- S4

Aggregation properties of CPDI-Ph in THF/water mixed solution with different f_w (Figure S3) -- S5

SEM images of (*S*)-CPDI-Ph aggregates in THF/water mixed solution (1.0×10^{-5} M) with f_w = 60 % after 10 s (Figure S4) --- S6

Time-dependent UV-visible, circular dichroism spectra, and SEM images of CPDI-Ph in THF/water mixed solution with f_w = 90 % (Figure S5) --- S7

Time-dependent UV-visible, circular dichroism spectra and SEM images of CPDI-Ph in THF/water mixed solution with f_w = 70 % (Figure S6) -- S8

PXRD results of CPDI-Ph nanomaterials (Figure S7) -- S9

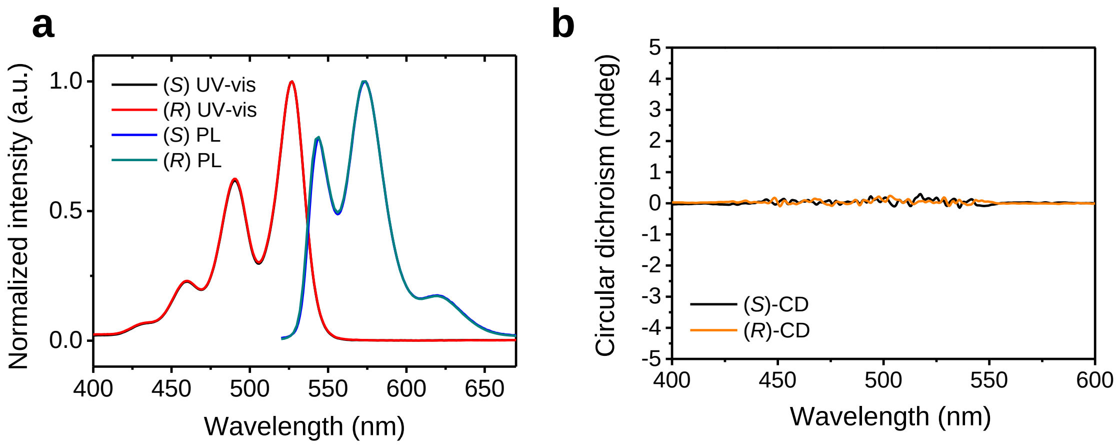

Figure S1. (a) UV-vis and PL spectra of CPDI-Ph in THF solution (1.0×10^{-5} M). (b) Circular dichroism spectrum of CPDI-Ph in THF solution (1.0×10^{-4} M).

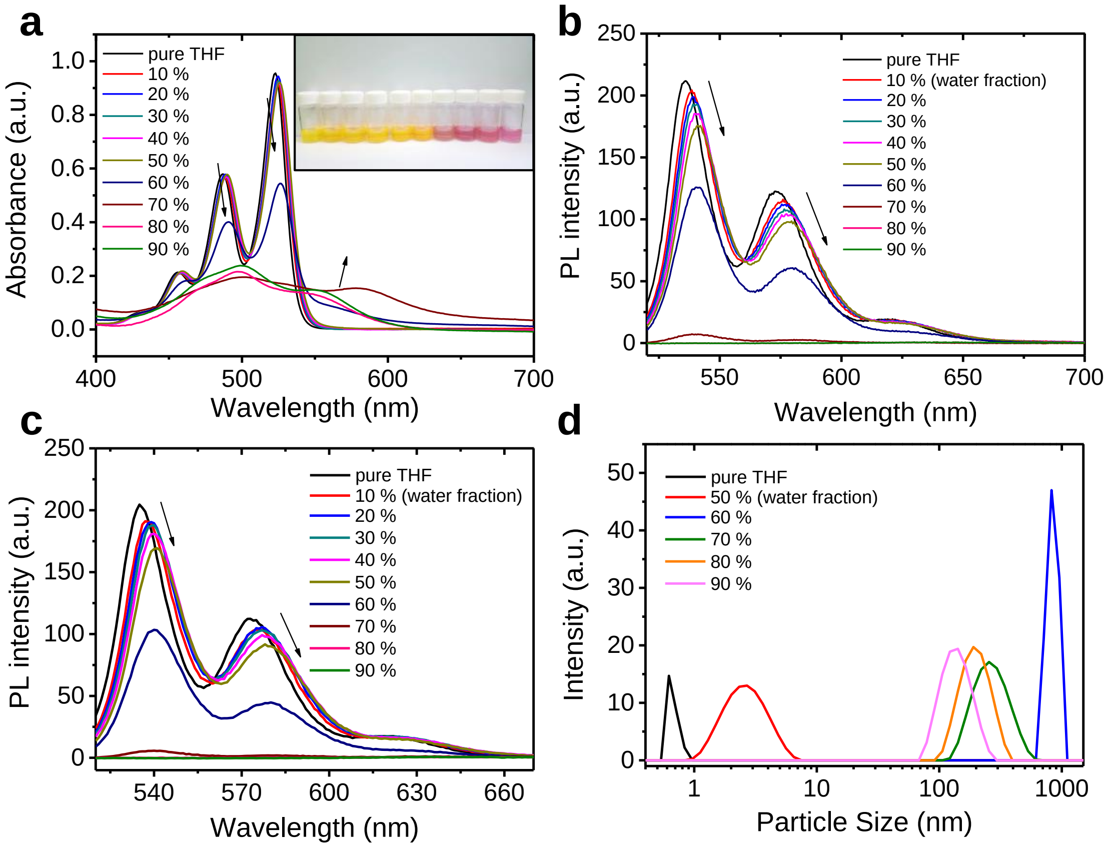

Figure S2. (a) UV-vis and (b) photoluminescence spectra of enantiomeric (*R*)-CPDI-Ph in THF/water mixed solution (1.0 $\times 10^{-5}$ M) with different f_w values. Inset of (a) shows the photograph images of THF solution (10^{-5} M) of CPDI-Ph at a different f_w (from left to right, f_w of 0% (i.e., pure THF) to 90% with 10% increment). (c) Photoluminescence spectra of enantiomeric (*S*)-CPDI-Ph THF/water mixed solution (1.0 $\times 10^{-5}$ M) with different f_w values. (d) Dynamic light scattering results of (*S*)-CPDI-Ph in THF/water mixed solution (1.0 $\times 10^{-5}$ M) with different f_w values (D_H = 0% : 0.619 nm, 50% : 2.61 nm, 60% : 823 nm, 70% : 257 nm, 80% : 192 nm, 90% : 134 nm).

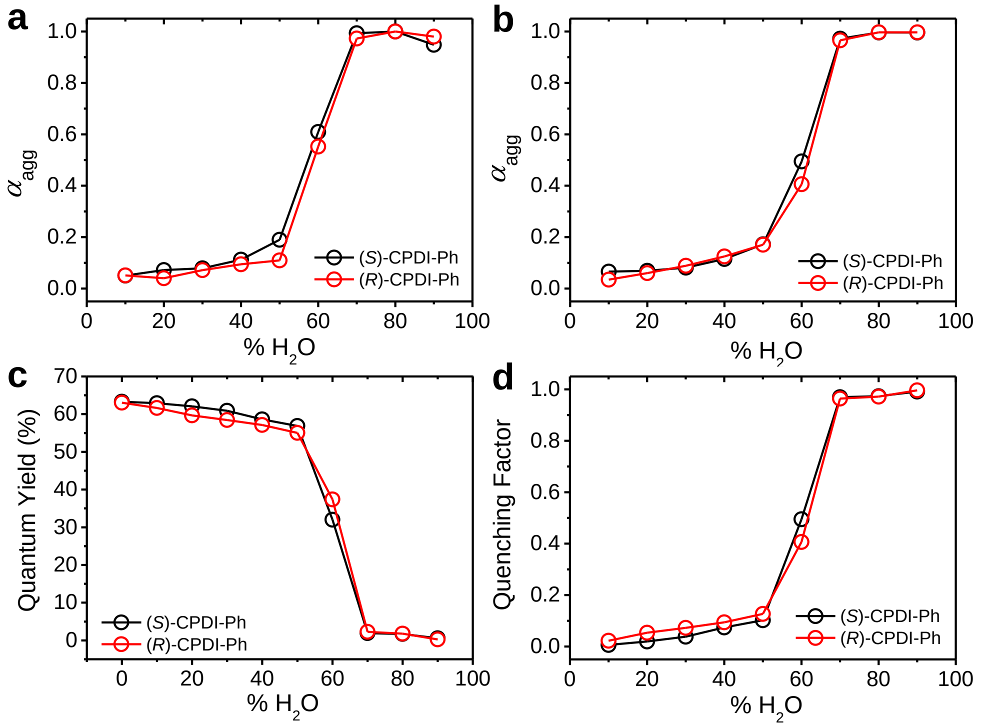

Figure S3. Plot of the degrees of aggregation with (a) UV-vis spectra, (b) PL spectra, (c) quantum yields, and (d) quenching factor for (*S*)- and (*R*)-CPDI-Ph in THF/water mixed solution (1.0 $\times 10^{-5}$ M) with different f_w.

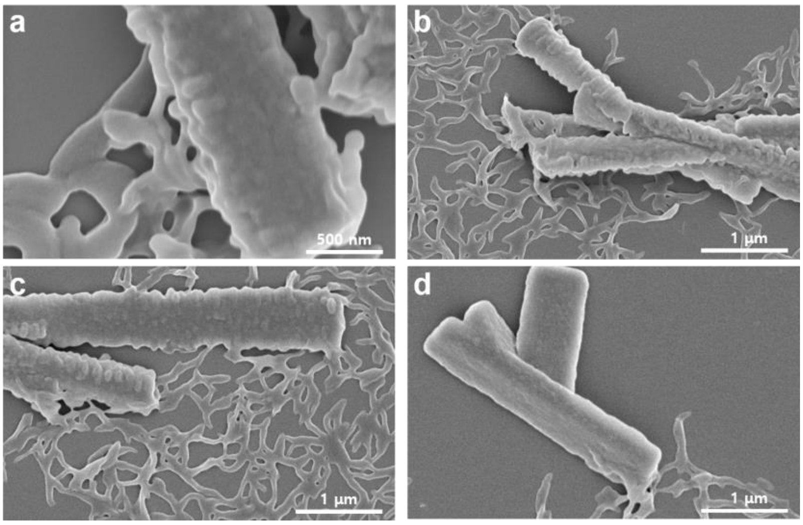

Figure S4. SEM images of (*S*)-CPDI-Ph aggregates in THF/water mixed solution (1.0×10^{-5} M) with f_w = 60 % after 10 s. (a) The magnified SEM image of Figure 2c. (b-d) SEM images of the different metastable nanobelts.

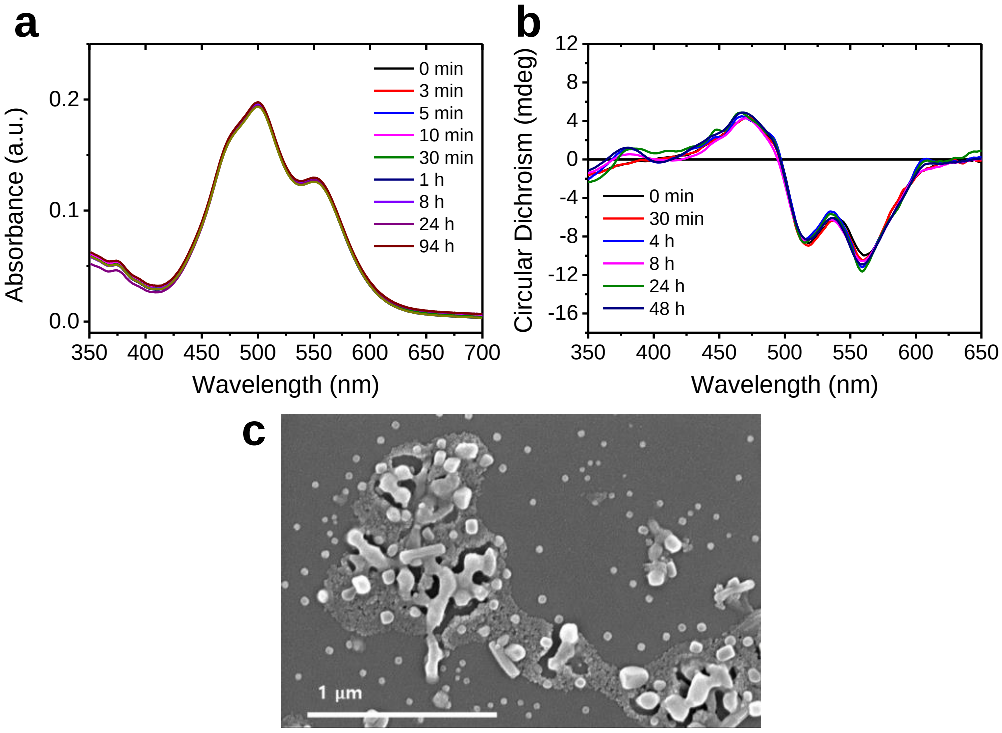

Figure S5. (a) Time-dependent UV-vis and (b) CD spectra of (*S*)-CPDI-Ph in THF/water mixed solution (1.0 ×10^{-5} M) with f_w = 90 %. (c) Time-dependent SEM images of (*S*)-CPDI-Ph in THF/water mixed solution (1.0 ×10^{-5} M) with f_w = 90 % after 4 h.

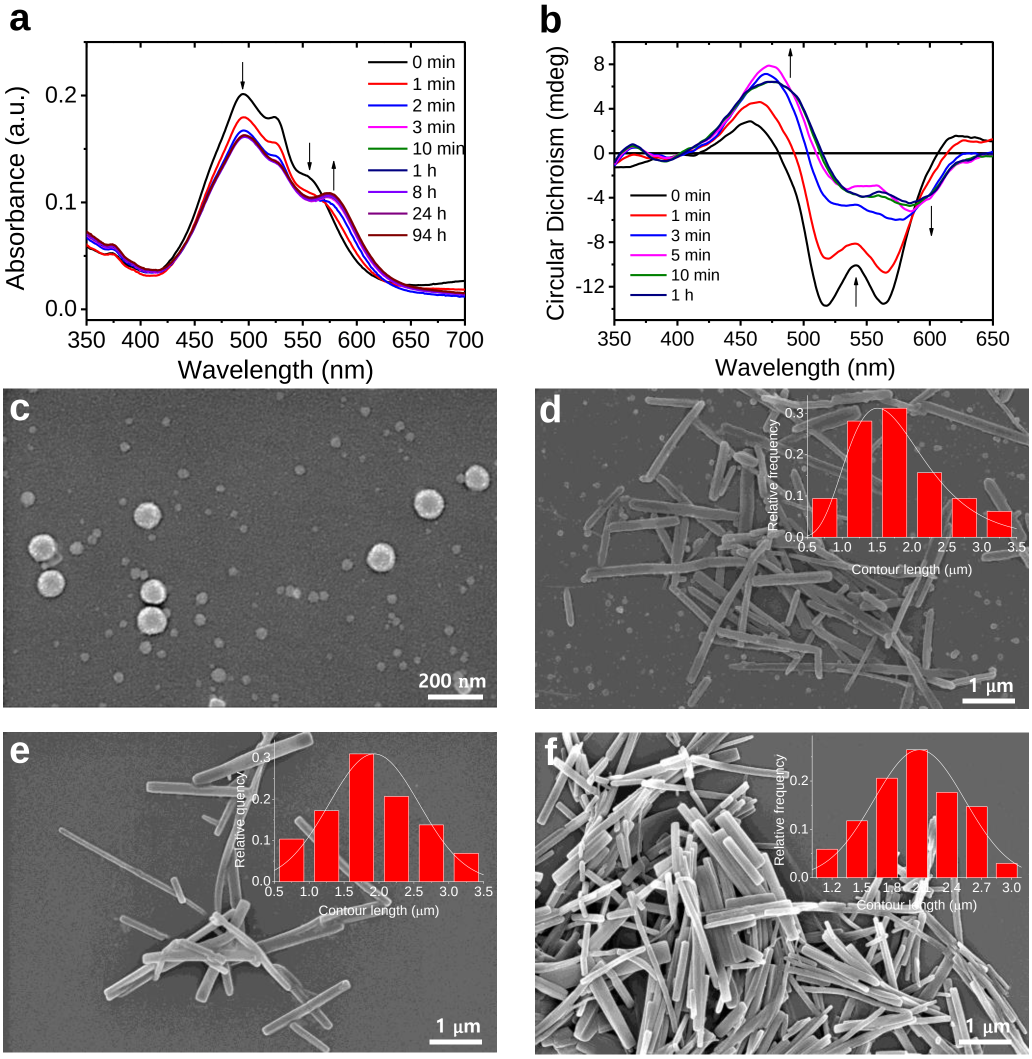

Figure S6. (a) Time-dependent UV-vis and (b) CD spectra of (*S*)-CPDI-Ph in THF/water mixed solution (1.0 ×10^{-5} M) with f_w = 70 %. Time-dependent SEM images of (*S*)-CPDI-Ph in THF/water mixed solution (1.0 ×10^{-5} M) with f_w = 70 % after (c) 10 s, (d) 5 min, (e) 4 h, and (f) 24 h.

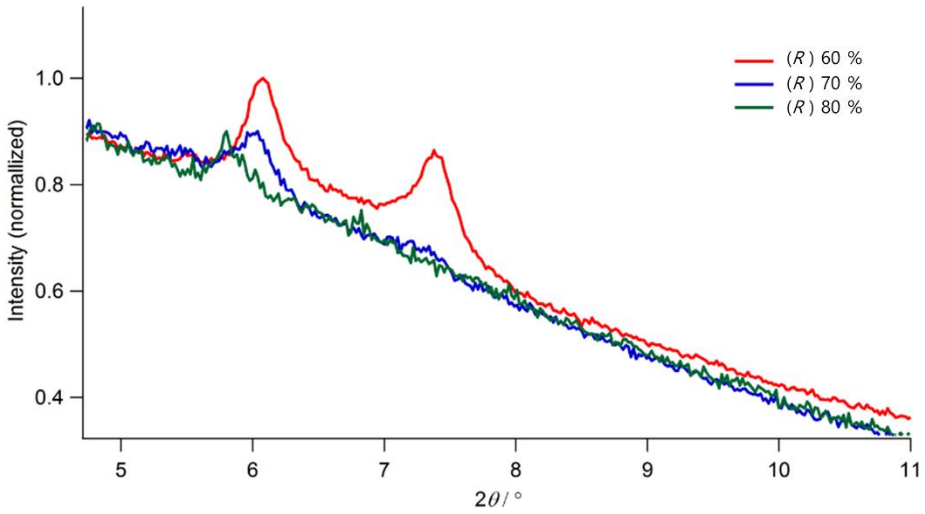

Figure S7. PXRD patterns of nanomaterials of (*R*)-CPDI-Ph in THF/water mixed solution (1.0 $\times 10^{-5}$ M) with f_w of 60% (red), 70% (blue) and 80% (green).
